# Field-Based Metabolomics of *Vitis vinifera L*. Stems Provides New Insights for Genotype Discrimination and Polyphenol Metabolism Structuring

**DOI:** 10.3389/fpls.2018.00798

**Published:** 2018-06-21

**Authors:** Kévin Billet, Benjamin Houillé, Thomas Dugé de Bernonville, Sébastien Besseau, Audrey Oudin, Vincent Courdavault, Guillaume Delanoue, Laurence Guérin, Marc Clastre, Nathalie Giglioli-Guivarc'h, Arnaud Lanoue

**Affiliations:** ^1^EA 2106 Biomolécules et Biotechnologie Végétales, Université de Tours, Faculté des Sciences Pharmaceutiques, Tours, France; ^2^Institut Français de la Vigne et du Vin Tours, France

**Keywords:** polyphenols, metabolomics, grape stems, metabotyping, *Vitis vinifera* L

## Abstract

Grape accumulates numerous polyphenols with abundant health benefit and organoleptic properties that *in planta* act as key components of the plant defense system against diseases. Considerable advances have been made in the chemical characterization of wine metabolites particularly volatile and polyphenolic compounds. However, the metabotyping (metabolite-phenotype characterization) of grape varieties, from polyphenolic-rich vineyard by-product is unprecedented. As this composition might result from the complex interaction between genotype, environment and viticultural practices, a field experiment was setting up with uniform pedo-climatic factors and viticultural practices of growing vines to favor the genetic determinism of polyphenol expression. As a result, UPLC-MS-based targeted metabolomic analyses of grape stems from 8 *Vitis vinifera* L. cultivars allowed the determination of 42 polyphenols related to phenolic acids, flavonoids, procyanidins, and stilbenoids as resveratrol oligomers (degree of oligomerization 1–4). Using a partial least-square discriminant analysis approach, grape stem chemical profiles were discriminated according to their genotypic origin showing that polyphenol profile express a varietal signature. Furthermore, hierarchical clustering highlights various degree of polyphenol similarity between grape varieties that were in agreement with the genetic distance using clustering analyses of 22 microsatellite DNA markers. Metabolite correlation network suggested that several polyphenol subclasses were differently controlled. The present polyphenol metabotyping approach coupled to multivariate statistical analyses might assist grape selection programs to improve metabolites with both health-benefit potential and plant defense traits.

## Introduction

Grapevine produces a myriad of bioactive phenolic compounds that are present in berries, leaves and stems, whereby this complex chemical composition can vary upon genotypes and environmental parameters. Because phenolic compounds in red wine have been associated to the “French paradox” (low incidence of cardiovascular disease despite a diet rich in saturated fats) an unprecedented interest was conferred to these natural products due to their potential benefit for human health (Smoliga et al., 2011). Indeed, numerous studies reported their role in preventing various diseases such as cancers, obesity, atherosclerosis and neurodegenerative diseases as well as an extension of cell longevity (Delmas et al., 2011; Weng and Yen, 2012).

**Figure 1:**
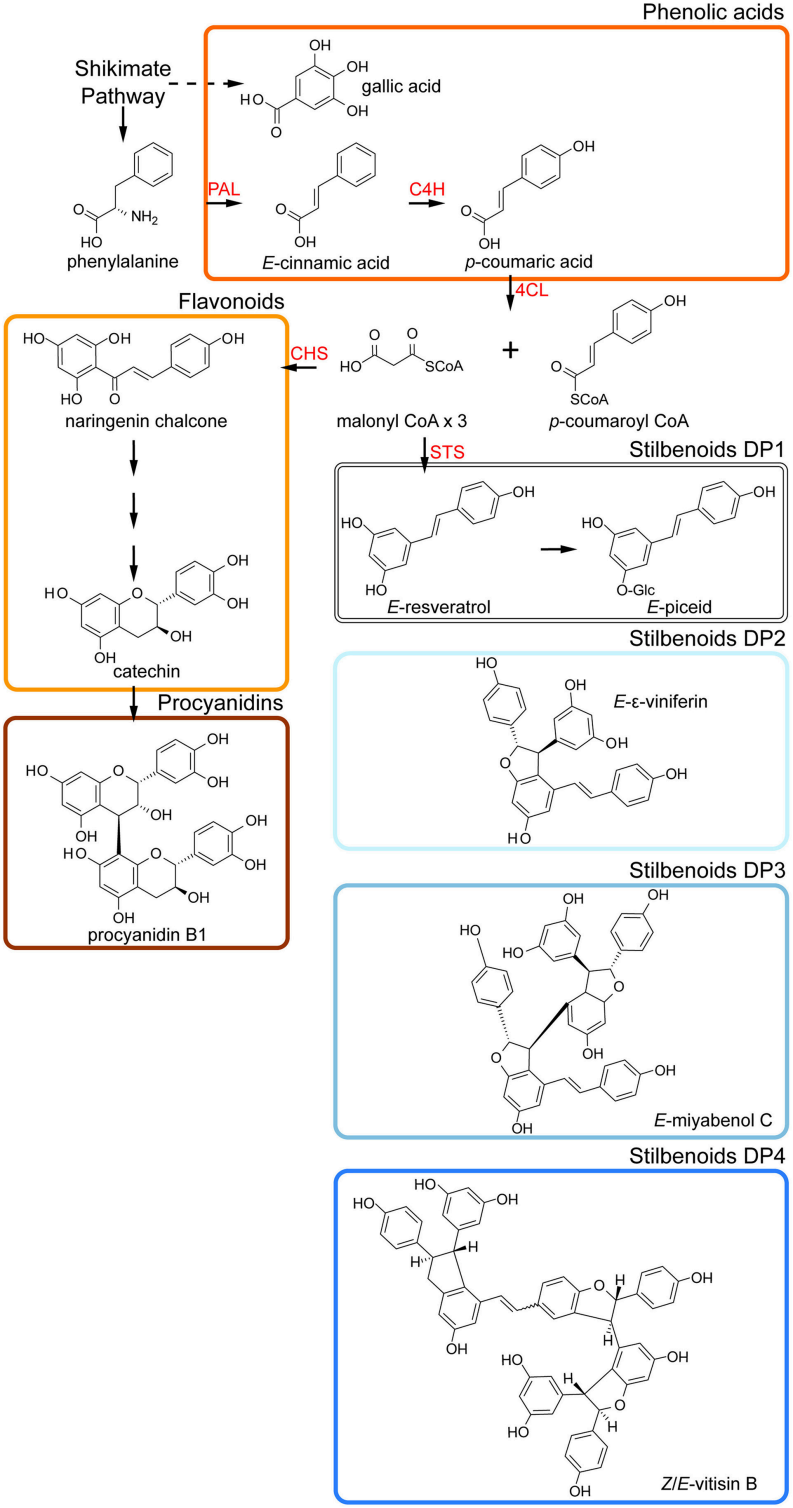
Overview of polyphenol metabolism in grape stems. PAL, phenylalanine ammonia lyase; C4H, cinnamate 4-hydroxylase; C3H, 4-coumarate 3-hydroxylase; 4CL, coumaric acid CoA ligase; CHS, chalcone synthase; STS, stilbene synthase.

Phenolic compounds represent a large class of specialized metabolites ubiquitous in the plant kingdom that derived from the phenylpropanoid and polyketide pathway. They are involved in many physiological functions such as plant cell wall structures, protection against UV radiation, defense against herbivores and pathogens and attraction of plant pollinators as floral pigments and flavors. Besides ubiquitous phenolic compounds including flavonoids, hydroxycinnamates, and benzoates, grapevine accumulated peculiar non-flavonoid compounds called stilbenoids. *E*-resveratrol formed by the stilbene synthase (STS) constitutes the first stilbenoid product (Figure 1) that is also the precursor for a wide range of stilbene biosynthetic products with diverse biological activities (Shen et al., 2009; Rivière et al., 2012). Stilbenoid compounds act as phytoalexines i.e locally induced antimicrobial molecules. *E*-resveratrol and its dimer *E*-ε-viniferin are biologically active against the grape pathogens downy mildew (Pezet et al., 2004), powdery mildew (Schnee et al., 2008) and gray mold (Adrian and Jeandet, 2012). Recently, resveratrol oligomers showed activities against grape fungal diseases (Lambert et al., 2013; Schnee et al., 2013). Grapevine accumulates stilbenoids mainly in lignified organs such as stems, seeds, roots and in clusters (Bavaresco et al., 1997; Bavaresco and Fregoni, 2001). By the way, the winter-pruned wood from vineyard has been proposed as a source of valuable bioactive compounds.

Metabolomics is the systematic study of all small-molecular weight metabolites present within a cell, tissue or organism. Grape metabolomics has contributed greatly to the chemical characterization of wine (Flamini et al., 2013). Advanced metabolomic tool applied to wine chemical analysis revealed unexpected signature, like the geographic origin of cooperage oak wood used during winemaking (Gougeon et al., 2009) and ancient winemaking practices (Jeandet et al., 2015). Metabolomics studies based on vineyards experiments have been mainly focused on berries during development and Botrytis infection (Zamboni et al., 2010; Agudelo-Romero et al., 2015; Negri et al., 2017; Wang et al., 2017). In grape stems, Lambert et al. (2013) reported that major compounds stilbenoids greatly vary with cultivars. In contrast, the targeted metabolomic analysis of polyphenol-rich grape stems is unprecedented. Recently, the development of metabolomic approaches has led to the concept of metabolic phenotype that considers a metabolic profile as a result of the genotype expression under environmental conditions. It is particularly relevant for plant metabotypes based on specialized metabolites since they might vary according biotic and abiotic stresses (Semmar et al., 2008).

The aim of the study was to assess to the variability of polyphenol metabolism across eight different varieties of *Vitis vinifera* L. (Sauvignon, Chenin, Chardonnay, Pinot Noir, Grolleau, Gamay, Malbec, and Cabernet Franc) grown under uniform environmental conditions (soil, microclimate, and training system) to emphasize the genetic dominance in polyphenol profiles. We used UPLC-MS-based targeted metabolomics to identify the metabolic composition of grape stem that correspond to the main site of accumulation for stilbenoids in vines. Chemometric tools including principal component analysis (PCA), partial least square discriminant analysis (PLS-DA) and hierarchical cluster analysis (HCA) were used to identify the overall metabolomic similarity/dissimilarity among the samples. Metabolomic and SSR-based clusterings were compared to assess the relationship between biochemical and genetic distances. In addition, pair wise correlation of metabolites highlighted new relationships within the stilbenoid metabolism.

## Materials and methods

### Vineyard features and environmental parameters

**Figure 2:**
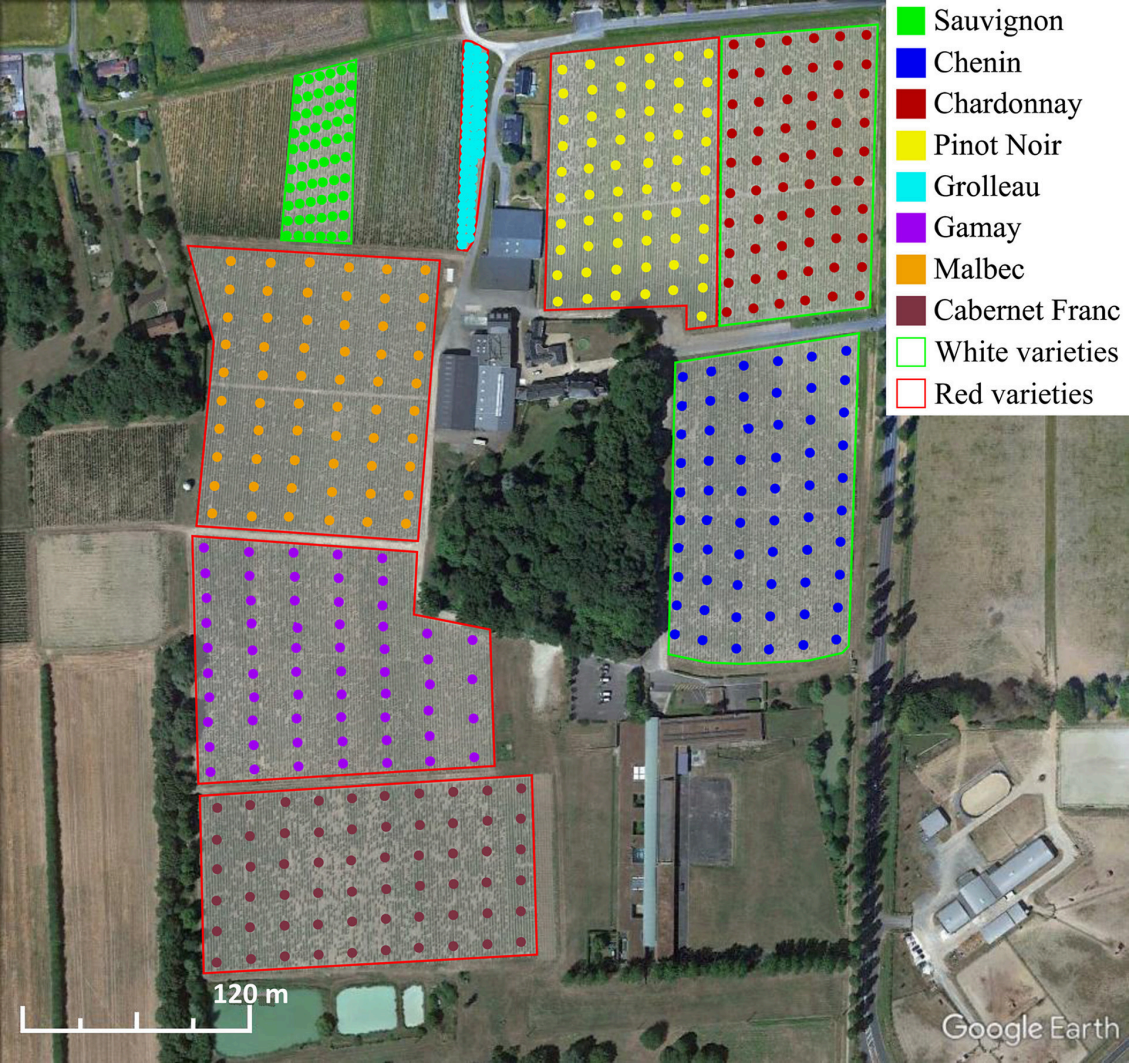
Location of the vineyard plots planted with the 8 different grape varieties in Amboise (Loire Valley, France). Grape stems were randomly pruned across the total area of each plot as indicated by circle positions.

The study was conducted during vintages 2013 and 2017 on 39-year-old vines in the school of Viticulture of Amboise in the Loire Valley Region (France; 47°39′59.46″N, 0°97′61.54″E). Eight adjacent vineyard parcels were planted with *V. vinifera* cv. Sauvignon, Chenin, Chardonnay, Pinot Noir, Grolleau, Gamay, Malbec (Cot), and Cabernet Franc (Figure 2; Table 1) on a clay-calcareous soil at a spacing of 1 m (within row) × 1 m (between rows) corresponding to 10,000 vines/ha. The vineyards were conducted with standard organic management practices with double Guyot pruning system. Climate in the Loire Valley is described as a temperate oceanic climate with, average daily temperatures from May to September 2013 corresponding to 17°C with a maximum of 34.8°C and a minimum of 3.4°C. Rainfall during this period corresponded to 297 mm with 70 rainy days. In 2017, for the same period, the average daily temperatures corresponded to 18°C with a maximum of 36.9°C and a minimum of 3.8°C. Rainfall during this period corresponded to 258 mm with 66 rainy days. Following the vegetative period and the grape harvest, stems from the eight parcels were pruned on 14 December 2013 and 19 December 2017.

**Table 1:** Description of the experimental setup in vineyards.

**Cultivar**	**Clone**	**Rootstock**	**Age (year 2013)**	**Space within row**	**Space between rows**	**Soil**	**Surface (ha)**	**GPS coordinates**
Sauvignon	cl 241	101-14	39	1 m	1 m	Calcareous clay	0.28	47°23'47.16“N 0°58'31.59”E
Chenin	cl 15	Riparia	39	1 m	1 m	Calcareous clay	1.62	47°23′44.49″N 0°58′44.20″E
Chardonnay	cl 75	SO4	39	1 m	1 m	Calcareous clay	1.29	47°23′46.80″N 0°58′44.95″E
Pinot noir	cl 668	SO4	39	1 m	1 m	Calcareous clay	1.36	47°23′45.90″N 0°58′37.81″E
Grolleau	cl 365	101-14	39	1 m	1 m	Calcareous clay	0.07	47°23′48.76″N 0°58′35.87″E
Gamay	cl 428	3309	39	1 m	1 m	Calcareous clay	1.77	47°23′40.95″N 0°58′32.30″E
Malbec	cl 1716	Riparia	39	1 m	1 m	Calcareous clay	1.84	47°23′46.24″N 0°58′30.49″E
Cabernet Franc	cl 623	101-14	39	1 m	1 m	Calcareous clay	1.63	47°23′37.26″N 0°58′35.65″E

### Extraction, analysis, and identification of metabolites

To overcome intra-plot variations, 60 stalks per parcel were pruned from 60 vines across the total area of each plot by harvesting an equal number of stalks from each vine row (Figure 2). One section of 10 cm long was cut from each stalk and then, we performed a randomized draw to constitute 5 pseudo-biological replicates for each grape variety where each lot was randomly composed by 10 sections (10 sections × 5 replicates × 8 varieties). Grape stems were stored for 10 weeks at 20°C in the dark to allow the post-pruned biosynthesis of stilbenoids (Houillé et al., 2015a). Grape stems were ground twice as previously described to obtain a powder with an average particle size of 1 mm. Stilbenoid extraction was adapted from Karacabey and Mazza (2008). Fifty milligrams of lyophilized powder were extracted in 1 mL ethanol/water solution (60:40; v/v) and shaken for 30 min at 1,400 rpm at 83°C and centrifuged at 18,000 x *g* for 5 min. The extracts diluted 1:5 with 0.1% formic acid were stored at −20°C prior to further analyses. UPLC-MS was performed using an ACQUITY™ Ultra Performance Liquid Chromatography system coupled to a photo diode array detector (PDA) and a Xevo TQD mass spectrometer (Waters, Milford, MA) equipped with an electrospray ionization (ESI) source controlled by Masslynx 4.1 software (Waters, Milford, MA). Analyte separation was achieved by using a Waters Acquity HSS T3 C18 column (150 × 2.1 mm, 1.8 μm) with a flow rate of 0.4 mL min^−1^ at 55°C. The injection volume was 5 μL. The mobile phase consisted of solvent A (0.1% formic acid in water) and solvent B (0.1% formic acid in acetonitrile). Chromatographic separation was achieved using an 18-min linear gradient from 5 to 60% solvent B. MS detection was performed in both positive and negative modes. The capillary voltage was 3,000 V and sample cone voltages were 30 and 60 V. The cone and desolvation gas flow rates were 60 and 800 Lh^−1^. Identification of analytes was based on retention times, m/z values, and UV spectra and by comparison with commercial standards, own purified compounds or data from literature when no authentic standards were available. Extraction and UPLC-MS analyses were performed in triplicates on the two independent experiments (vintages 2013 and 2017).

### Data mining

First, a list of mass-to-charge (m/z) ratios corresponding to molecular ions was manually established based on total ion current chromatograms from both positive and negative modes from the eight cultivars (Table 2). Secondly, UPLC-MS analyses were achieved using selected ion monitoring (SIM) mode and the resulting SIM chromatograms were integrated using the subroutine QuanLynx 4.1 for data mining. Peak integration was performed using the ApexTrack algorithm with a mass window of 0.1 Da and relative retention time window of 1 min followed by Savitzky–Golay smoothing (iteration = 1 and width = 1). The resulting pairs of m/z values and retention times were also manually examined. To evaluate the robustness of measurements and analytical variability, a pool of samples was prepared from grape stems of all varieties to obtain a quality control sample (QC) and the samples were randomly injected independently from the genotype status. Three QC samples were injected to equilibrate the system before each analytical sample set and one QC sample was injected every 8 samples to check for potential analytical drift. QC samples were analyzed by Principal Component Analysis to evaluate the reproducibility of the UPLC-MS method (Fiehn et al., 2008).

**Table 2:** List of grape stem polyphenols identified in studied cultivars.

**Peak**	**RT (min)**	**Phenolic class**	**Compound assignment**	**m/z [M-H]-**	**Product ions (ES-)**	**m/z [M+H]+**	**λmax (nm)**	**References**
1	1.69	Phenolic acid	Gallic acid	169	163, 125		269	Sun et al., 2007
2	9.65	Stilbenoid DP1	*E*-resveratrol	227	183, 143	229	305, 317	Pawlus et al., 2012; Lambert et al., 2013
3	9.17	Stilbenoid DP1	*E*-piceatannol	243	185, 159	245	322	Pawlus et al., 2012; Lambert et al., 2013
4	5.38	Flavonoid	Catechin	289	261, 205	291	229, 278	Lambert et al., 2013
5	6.73	Flavonoid	Epicatechin	289	211, 152	291	229, 278	Lambert et al., 2013
6	4.12	Flavonoid	Gallocatechin	305	109	307	332, 369	Ehrhardt et al., 2014
7	8.43	Stilbenoid DP1	*E*-piceid	389	227		278.7	Ehrhardt et al., 2014
8	8.48	Flavonoid	Epicatechin 3-*O*-gallate^*^	441	289, 169	443	276.7	Ehrhardt et al., 2014
9	9.09	Flavonoid	Astilbin	449	303, 285		231, 289.7	Souquet et al., 2000
10	10.22	Stilbenoid DP2	*Z*-resveratrol dimer1^*^	453	277, 265	455	232sh, 279, 285	Püssa et al., 2006
11	13.39	Stilbenoid DP2	*Z*-resveratrol dimer2^*^	453	428	455	225, 282.7	Püssa et al., 2006
12	13.73	Stilbenoid DP2	*E*-ε-viniferin	453	411, 369	455	225sh, 323	Pawlus et al., 2012; Lambert et al., 2013
13	14.81	Stilbenoid DP2	*E*-ω-viniferin^*^	453	369, 263	455	225sh, 323	Püssa et al., 2006
14	15.36	Stilbenoid DP2	*E*-δ-viniferin^*^	453	227	455	225sh, 309	Pezet et al., 2003; Mulinacci et al., 2010
15	8.93	Stilbenoid DP2	Ampelopsin A	469	451, 316	471	281.7	Lambert et al., 2013
16	10.84	Stilbenoid DP2	Scirpusin A1^*^	469	451, 395	471	324.7	Kong et al., 2010; Moss et al., 2013
17	12.39	Stilbenoid DP2	Scirpusin A2^*^	469	379, 301	471	321.7	Kong et al., 2010; Moss et al., 2013
18	7.47	Stilbenoid DP2	Restrytisol A^*^	471	455, 377, 246		230, 276	Cichewicz et al. 2000; Jean-Denis et al., 2006; Mulinacci et al., 2010
19	8.32	Stilbenoid DP2	Restrytisol B^*^	471	455, 379, 349		235, 267	Cichewicz et al., 2000; Jean-Denis et al., 2006; Mulinacci et al., 2010
20	8.42	Stilbenoid DP2	Restrytisol 3^*^	471	455, 389, 227		234, 330	Cichewicz et al., 2000; Jean-Denis et al., 2006; Mulinacci et al., 2010
21	8.75	Flavonoid	Quercetin-3-*O*-glucuronide	477	301, 151	479	256, 354	Souquet et al., 2000; Ehrhardt et al., 2014
22	4.76	Procyanidin	Procyanidin B1	577	295, 162	579	280, 313	Ehrhardt et al., 2014
23	5.13	Procyanidin	Procyanidin B3^*^	577	295, 162	579	280, 313	Ehrhardt et al., 2014
24	6.18	Procyanidin	Procyanidin B4^*^	577	514		579	Ehrhardt et al., 2014
25	7.02	Procyanidin	Procyanidin B2		577		579	Ehrhardt et al., 2014
26	7.42	Procyanidin	Procyanidin B5^*^	577	522, 471		579	Ehrhardt et al., 2014
27	10.62	Stilbenoid DP2	Resveratrol dimer glycoside^*^	615	567, 537, 453		323	Moss et al., 2013
28	14.71	Stilbenoid DP3	α-viniferin^*^	677	423	679	230sh, 285	Mattivi et al., 2011
29	12.34	Stilbenoid DP3	Resveratrol trimer1^*^	679		681		Püssa et al., 2006; Moss et al., 2013
30	12.91	Stilbenoid DP3	Resveratrol trimer2^*^	679		383		Püssa et al., 2006; Moss et al., 2013
31	13.33	Stilbenoid DP3	Resveratrol trimer3^*^	679				Püssa et al., 2006; Moss et al., 2013
32	14.06	Stilbenoid DP3	*E*-miyabenol C	679	573, 345	681	284	Lambert et al., 2013
33	15.31	Stilbenoid DP3	Resveratrol trimer4^*^	679	653, 454	681	230sh, 322	Püssa et al., 2006; Moss et al., 2013
34	5.63	Procyanidin	Procyanidin trimer^*^	865	664, 576	867	279	Monagas et al., 2006
35	14.45	Stilbenoid DP4	Dehydrogenated resveratrol tetramer ^*^	904	679		295	Ito et al., 1999
36	12.17	Stilbenoid DP4	Hopeaphenol	905	811, 717, 705, 451, 359	907	281.6	Lambert et al., 2013
37	12.56	Stilbenoid DP4	Isohopeaphenol	905	811, 717, 451, 359	907	283.9	Lambert et al., 2013
38	13.06	Stilbenoid DP4	Resveratrol tetramer1^*^	905		907	285	Püssa et al., 2006; Flamini et al., 2013
39	13.56	Stilbenoid DP4	Resveratrol tetramer2^*^	905		907	284, 331	Püssa et al., 2006; Flamini et al., 2013
40	16.03	Stilbenoid DP4	Resveratrol tetramer3^*^	905	573	907	323	Püssa et al., 2006; Flamini et al., 2013
41	16.2	Stilbenoid DP4	*E*-vitisin B	905	799, 359	907	322	Pawlus et al., 2012; Lambert et al., 2013
42	12.15	Stilbenoid DP4	Viniferol E^*^	923				Fujii et al. (2005)

RT, retention time

* *tentative assignments based on MS data, UV spectra, elution order available from literature*.

### Statistical analysis

Multivariate Statistical Data Analysis (MVA) was performed using SIMCA P+ version 12.0 (Umetrics AB, Umeå, Sweden). All variables were mean-centered and unit-variance (UV) scaled prior to MVA. Principal Component Analysis was applied as unsupervised MVA method to reveal differences and similarities among samples without bias on experimental groups. Partial Least Squares Discriminate Analysis (PLS-DA) was used as supervised clustering to maximize the difference between metabolic phenotype and to better evaluate group structure. PLS-DA results are visualized as score plots, where each symbol represents one individual sample (grape stem extract) and the position of the symbol is achieved through a combination of this individual values on all variables. The variables that contribute to the separation of the classes can then be identified (Eriksson et al., 2006). The quality of the PLS-DA model was estimated with *R*^2^*X*(cum) the cumulative modeled variation in the X matrix (polyphenols), *R*^2^*Y*(cum) the cumulative modeled variation in the *Y* matrix (grape variety samples), and *Q*^2^(cum) the predictive ability. Hierarchical cluster analyses (HCA) using Ward's minimum variance method were carried out to identify sub group of cultivars. Sample harvests, UPLC-MS analyses, and MVA have been repeated twice independently (vintages 2013 and 2017.) To compare the two hierarchical clusterings obtained from the datasets of both polyphenols and SSR markers, we used the similarity index of Fowlkes-Mallows (B_*k*_) that is ranged between zero and one, where zero represented complete dissimilarity and one, complete equality (Fowlkes and Mallows, 1983). Calculations were made with R (v3.2.2; R Core Team, 2015) with the “dendextend” package (v1.1.0). Pair-wise correlations within the data matrix of 42 metabolites were calculated for all 861 metabolite pairs. Matrix of pairwise Pearson correlation coefficients were calculated in R with the “cor” function and significance of association were obtained with the “cor.test” function. Significant correlations (>0.5) were visualized using the “igraph” package (v1.0.1) (Csardi and Nepusz, 2006) and the “graph.adjacency” function which is specially designed for such matrices. Layout of the network was adapted with the Fruchterman-Reingold algorithm (Fruchterman and Reingold, 1991).

### Reagents and chemicals

*E*-Resveratrol, *E*-piceatannol, *E*-piceid, catechin, epicatechin, gallic acid, gallocatechin, astilbin, procyanidin B1, procyanidin B2, and quercetin-3-*O*-glucuronide were purchased from Sigma-Aldrich (St Louis, MI, USA). *E*-ε-viniferin, *Z*/*E*-vitisin B, ampelopsin A, *E*-miyabenol C, and hopeaphenol were obtained by previous extraction from grape stems (Houillé et al., 2014). Acetonitrile, methanol and formic acid were purchased from ThermoFisher Scientific (Courtaboeuf, France). Ultra-pure water was prepared with a Milli-Q water purification system (Millipore, USA).

### SSR selection

Twenty two SSR markers previously developed for grape genotyping were selected for this study, corresponding to 20 SSR markers proposed by Lacombe et al. (2013) and 2 additional markers VrZAG62 and VrZAG79 from Sfec et al. (1999). The present list of markers includes the 9 SSR markers proposed by The European Vitis Database (Maul et al., 2012).

## Results

### Concentrations of major stilbenoids vary among the selected genotypes

Vineyards were chosen in the Loire Valley region, the third largest wine producing area in France. To minimize the effect of environmental conditions, we selected adjacent vineyard plots having the same calcareous clay soil, each planted with different grape varieties of the same age (Sauvignon, Chenin, Chardonnay, Pinot Noir, Grolleau, Gamay, Malbec, and Cabernet Franc; Figure 2; Table 1). During the grape vine dormancy, grape stems from the eight parcels were randomly pruned across the total area to overcome intra-plot variation and stored as previously described to allow post-pruned stilbenoid accumulation (Houillé et al., 2015a; Billet et al., 2018). An accurate quantification of the major stilbenoids was achieved based on calibration curves with pure stilbenoid standards. The major stilbenoids accumulated in the eight analyzed cultivars were *E*-resveratrol, *E*-ε-viniferin, ampelopsin A, and hopeaphenol whereas other analyzed stilbenoids (*E*-piceatannol, isohopeaphenol, *E*-vitisin B, and *E*-miyabenol C) accounted for only a minor part of the total stilbenoids (Table S1). Our results and previous studies provided striking evidence that stilbenoid composition is highly variable in grape stems and particularly depends on genotype (Gatto et al., 2008; Lambert et al., 2013; Pawlus et al., 2013). However, these studies are restricted to few major stilbenoids representing only partial fingerprints and a systematic study of the global polyphenol composition would better assess the relationship between metabolomic composition and genotype.

### Establishing the grape stem metabolome

To identify polyphenols in grape stem extracts, qualitative UPLC-MS analyses in both ESI^+^ and ESI^−^ modes were carried out on each grape variety and a list of molecular ions was established (Table 2). On a total of 42 compounds, 17 analytes were annotated and their molecular assignment confirmed by external standards i.e., *E*-Resveratrol, *E*-piceatannol, *E*-piceid, catechin, epicatechin, gallic acid, gallocatechin, astilbin, procyanidin B1, procyanidin B2, quercetin-3-*O*-glucuronide, *E*-ε-viniferin, *E*-vitisin B, ampelopsin A, *E*-miyabenol C, hopeaphenol, and isohopeaphenol. The remaining components were provisionally assigned according to MS data, UV spectra, and elution order obtained from the literature (Table 2). For instance, the peak 8 with an [M-H]^−^ ion at m/z 441 formed daughter ions at m/z 289 (catechin unit) and 169 (gallate unit) and was putatively assigned to epicatechin 3-*O*-gallate (Ehrhardt et al., 2014). Among resveratrol-resveratrol homodimers ([M-H]^−^ 453), two compounds (peaks 10 and 11) were eluted before the well-known *E*-ε-viniferin as well as two additional ones after (peaks 13 and 14). Compounds 13 and 14 were temporary attributed to *E*-ω-viniferin and *E*-δ-viniferin in accordance with the elution profile described in the literature (Pezet et al., 2003; Püssa et al., 2006; Mulinacci et al., 2010). Among the resveratrol-piceatannol heterodimers ([M-H]^−^ 469), two analytes (peaks 16 and 17) were eluted after the well characterized ampelopsin A and were tentatively identified as two structural isomers of scirpusin A (Kong et al., 2010; Moss et al., 2013). Three compounds with m/z 471 (peaks 18–20) were detected, all producing the same fragmentation pattern including the product ion m/z 455 (−16 Da, loss of monoxide) and m/z 349 (−122 Da, consecutive loss of carbon monoxide and phenol). These three compounds corresponding to oxidized resveratrol dimers were temporary assigned to restritysol A or B and an additional isomeric form (Cichewicz et al., 2000; Jean-Denis et al., 2006; Mulinacci et al., 2010). A group of five compounds with m/z 577 (peaks 22–26) were attributed to B-type procyanidins (Ehrhardt et al., 2014). Through comparison with external standards, peak 22 and 25 were identified as procyanidin B1 and B2, respectively. The peak 27 with an [M-H]^−^ ion at m/z 615 produced a product ion at m/z 453 (a resveratrol dimer) corresponding to a loss of glucose (162 Da). Consequently, this compound was attributed to a resveratrol dimer glucoside in agreement with Moss et al. (2013). The peak 28 with an [M-H]^−^ ion at m/z 677 corresponding to a resveratrol dehydrotrimer was temporary assigned to α-viniferin (Mattivi et al., 2011). A total of 5 peaks (29–33) exhibited an [M-H]^−^ ion at m/z 679 including the known *E*-miyabenol C (peak 32) were assigned to resveratrol trimeric forms. Due to their low concentration in grape stems no fragmentation pattern could be obtained. The peak 34 with an [M-H]^−^ ion at m/z 865 produced a product ion at m/z 576 corresponding to the loss of a catechin unit (289 Da). Therefore, this compound was assigned to procyanidin trimer (Monagas et al., 2006). The compound with m/z 904 was temporary assigned to a dehydrogenated resveratrol tetramer according to Ito et al. (1999). A group of six compounds (peaks 36–41) exhibiting an [M-H]^−^ ion at m/z 905 including hopeaphenol (peak 36), isohopeaphenol (peak 37), and *E*-vitisin B (peak 41) were assigned to resveratrol tetrameric forms. Additionally, a compound producing an ion at m/z 923 (peak 42) was temporary assigned to viniferol E according to Fujii et al. (2005).

### Analysis of metabolomic variations with multivariate statistical analysis enables genotype discrimination

**Figure 3:**
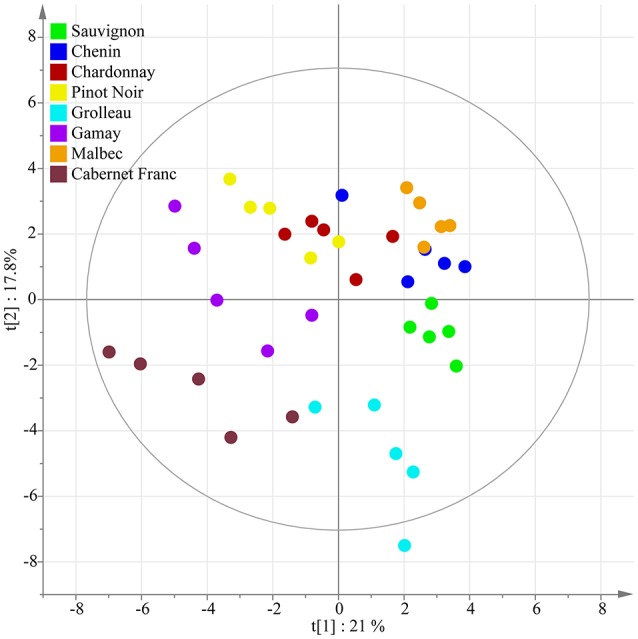
Unsupervised classification using PCA. Score plot on polyphenol data from grape stems of eight genotypes in 2013. Variables in plot were colored according to the genotype.

**Figure 4:**
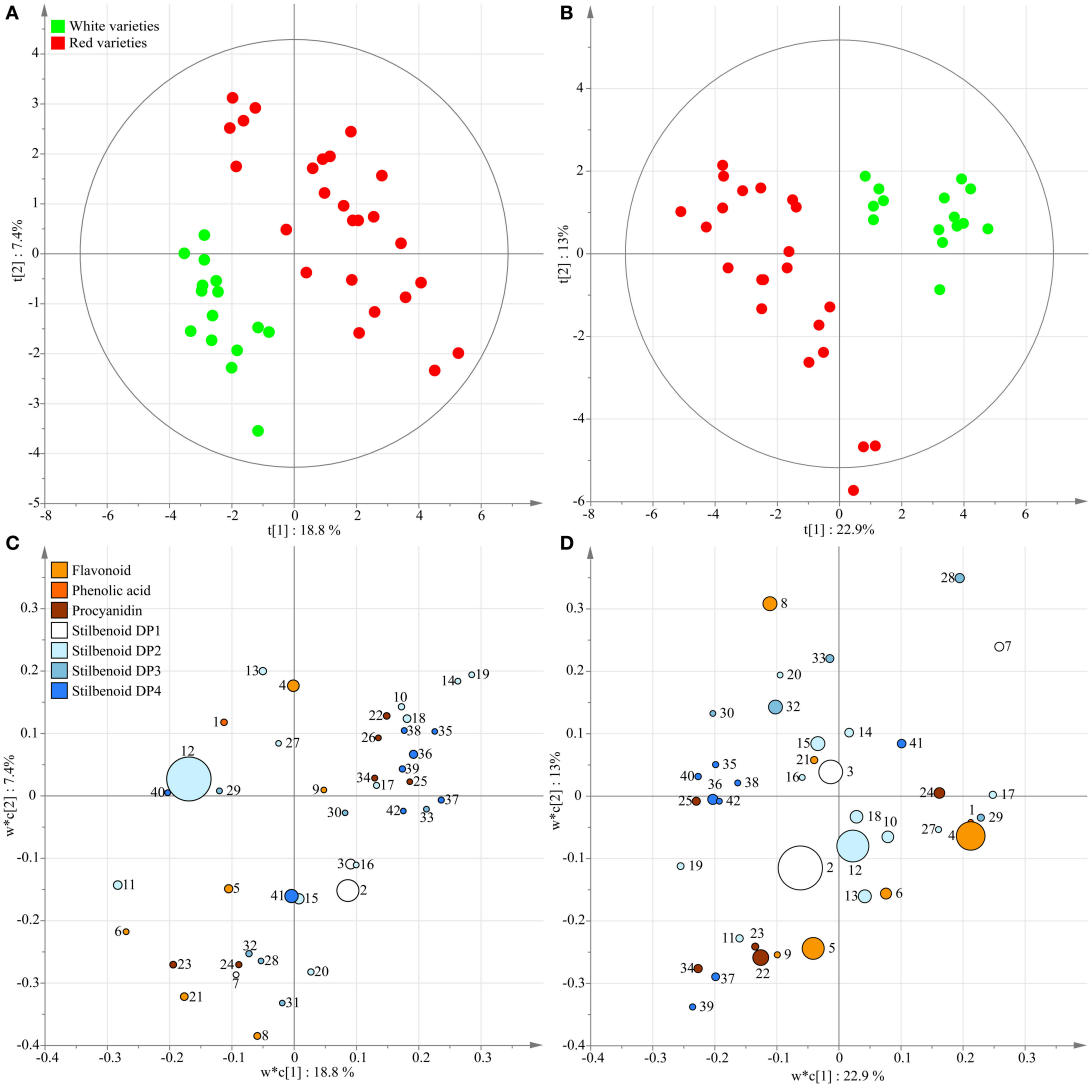
Supervised classification using PLS-DA with “cultivar color” as dependent variable on polyphenol data from grape stems of eight genotypes in 2013 **(A,C)** and 2017 **(B,D)**. Variables in score plots **(A,B)** were colored according to cultivar color. Variables in the loading plots, **(C,D)** were colored according to polyphenol class with round size relative to the abundance of the selected ion. Numbers indicate the ID of the compounds as given in Table 2.

A principal component analysis was performed as unsupervised clustering to show similarities and differences among the polyphenol dataset from 2013. The PCA score plot of the two first components shown in Figure 3 explained 38.8% of the variation. Although a perfect separation of sample groups could not be obtained, differences could be identified according to the genotypic origin of grape stems and to the grape varietal color. Indeed, the three white cultivars Chenin, Sauvignon, and Chardonnay were regrouped on the PCA score plot. Then, a PLS-DA approach was applied to sharpen group separation and to predict a dependent variable such as cultivar color or genotype. PLS-DA score plot with cultivar color as variable (diagnostic of the model: *R*^2^X_cum_ = 56%, *R*^2^Y_cum_ = 93%, ${\text{Q}}_{\text{cum}}^{2}$ = 64%) clearly clustered two classes (Figure 4A) with PC1 and PC2 explaining 18.8 and 7.4% of the variation, respectively. The loading plot shows the underlying metabolites responsible for the separation with round size relative to the abundance of the selected ion (Figure 4C). The two most abundant ions *E*-ε-viniferin (peak 12) and *E*-resveratrol (peak 2) were not responsible for the group separation according to cultivar color, however some minor compounds participate to color discrimination, e.g., DP4 stilbenoids (peak 35–39, 42) and procyanidins (peak 22, 25, 34), in red cultivars with PC1 positives scores. PLS-DA conducted in year 2017 (*R*^2^X_cum_ = 52%, *R*^2^Y_cum_ = 97%, ${\text{Q}}_{\text{cum}}^{2}$ = 77%) showed similar results (Figures 4B,D).

**Figure 5:**
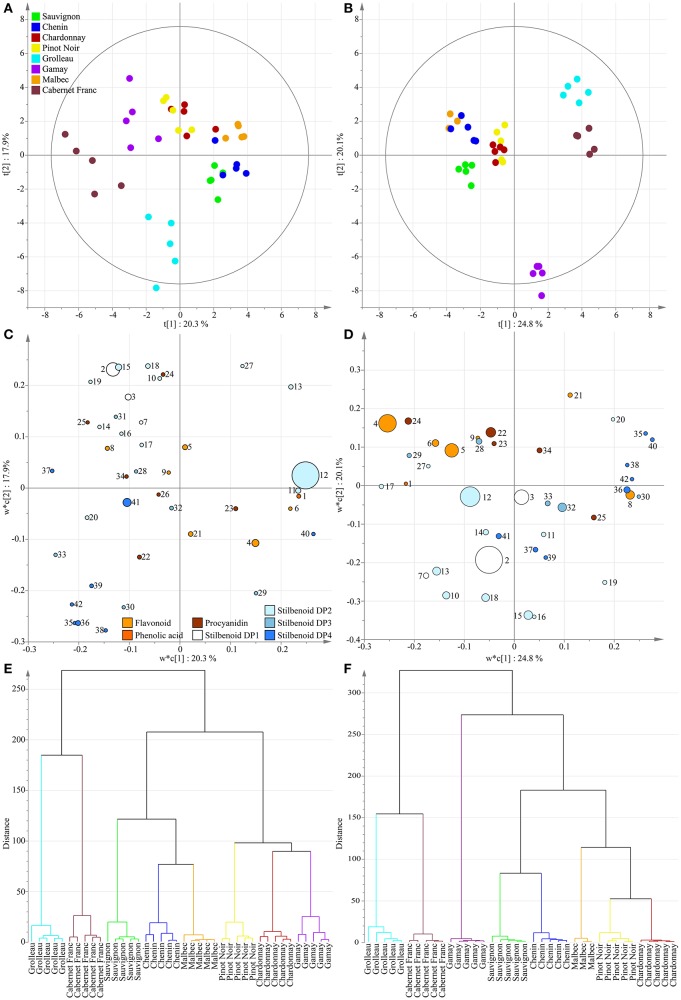
Supervised classification using PLS-DA with “genotype” as dependent variable on polyphenol data from grape stems of eight genotypes in 2013 **(A,C,E)** and 2017 **(B,D,F)**. Score plots **(A,B)**, PLS-DA loading plots **(C,D)**, and dendrograms of HCA **(E,F)** using Ward's minimum variance method for the loading matrix. Variables in score plot were colored according to genotype. Variables in the loading plot were colored according to polyphenol class with round size relative to the abundance of the selected ion. Numbers indicate the ID of the compounds as given in Table 2.

In a second step, a PLS-DA with genotype as dependent variable was performed (*R*^2^X_cum_ = 82%, *R*^2^Y_cum_ = 90%, ${\text{Q}}_{\text{cum}}^{2}$ = 74%). The score plot explained 38.2% of the total variation (Figure 5A) and the corresponding loading plot showed the contribution of each polyphenol (Figure 5C). No single polyphenol (biomarker) or specific class of polyphenol was responsible for the varietal discrimination albeit some metabolites were found prominent in specific genotypes. *E*-ε-viniferin-rich varieties (Chenin, Sauvignon, Malbec) are projected in PC1 positive whereas stilbenoid DP4-rich varieties (Cabernet Franc, Grolleau) are projected in PC1 and PC2 negative scores. *E*-resveratrol-rich varieties e.g., Gamay are projected in PC2 positive whereas *E*-resveratrol-poor varieties e.g., Grolleau are projected in PC2 negative. Such PLS-DA was reproduced on the polyphenol dataset in year 2017 (*R*^2^X_cum_ = 89%, *R*^2^Y_cum_ = 95%, ${\text{Q}}_{\text{cum}}^{2}$ = 80%) and the same metabolic signatures were observed (Figures 5B,D). A hierarchical clustering analysis (HCA) was applied on the loading matrix to identify the differential variables that account for the separation. A dendrogram showing the overall structural similarity determined by Ward's clustering based on Euclidean distance is represented in Figure 5E (year 2013) and Figure 5F (year 2017). Interestingly, HCA shows a perfect separation of classes as previously suspected on Figures 5A,B and enables the varietal discrimination of grape stems based on polyphenol-targeted metabolomics coupled to MVA. Moreover, the dendrogram structures (Figures 5E,F) reveal subgroups of cultivars suggesting the likelihood of distance in between the different metabolic phenotypes that were just established.

### Testing relationship between metabolomic and genetic distance among the eight grape varieties

**Figure 6:**
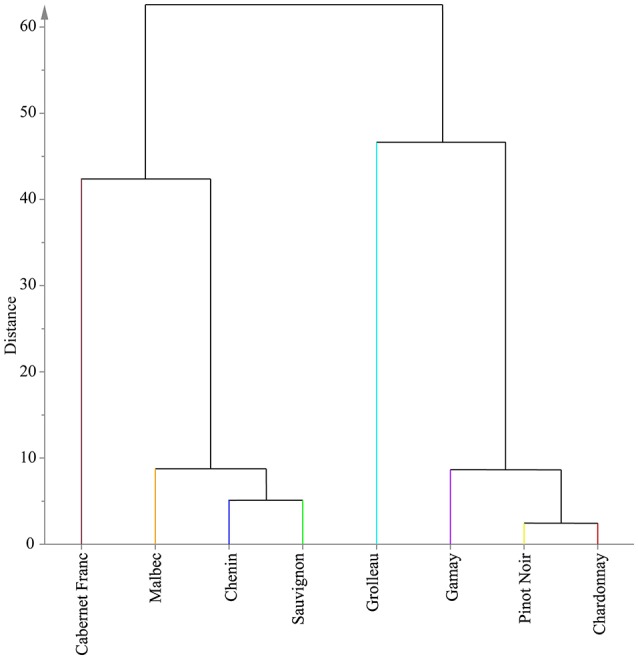
Dendrogram of HCA based on Ward's clustering of 22 SSR markers of the eight genotypes.

**Figure 7:**
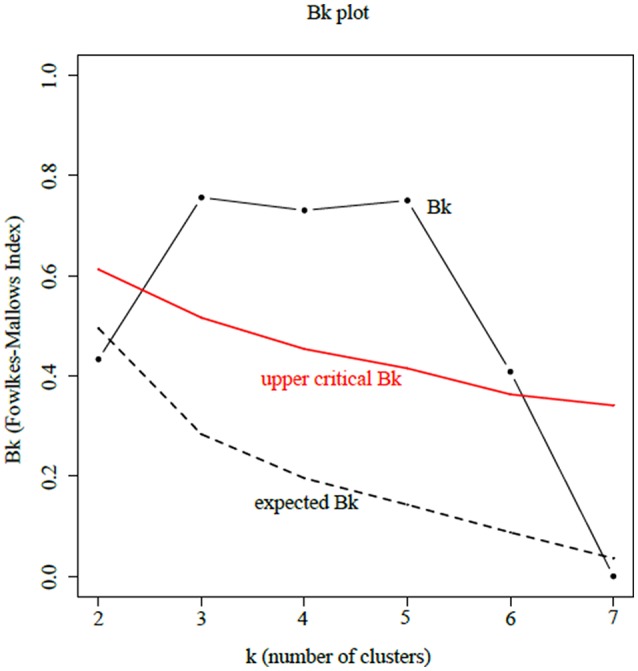
Similarity index of the two dendrograms obtained from polyphenols in 2013 and SSR markers as function of *k* clusters selected.

Since the field setup was chosen to mitigate the environmental impact on polyphenol metabolism, we hypothesized that a genetic determinism was responsible for the biochemical distance described above. To support this assumption, we used 22 Simple Sequence Repeats (SSRs) previously characterized by Lacombe et al. (2013) and Sfec et al. (1999) for genotyping of the grapevine cultivars to assess the link between metabolic phenotype and genetic variation. HCA was applied on the loading matrix containing the 22 SSRs of the eight cultivars and the corresponding dendrogram is presented in Figure 6. Clustering based on SSRs markers showed relative similarities with that on polyphenols in year 2013 (Figure 5E). Particularly, the sub group containing Gamay, Pinot Noir, and Chardonnay was observed for both types of datasets. Chenin, Sauvignon, and Malbec were also found to be closely related. To refine the similarity study between the two dendrograms, we used the Fowlkes-Mallows index (*B*_*k*_). The *B*_*k*_ index relies on the *k* parameter that represents the threshold at which both dendrograms are cut and the number of matching entries is count in the resulting *k* clusters of each tree (Fowlkes and Mallows, 1983). The resulting *B*_*k*_ plot presented in Figure 7 showed excellent similarity index for cluster numbers *k* between 3 and 5. This indicates that the structure clustering of both polyphenol and SSR marker datasets are closely related and polyphenol composition in grape stems was linked to the genetic distance of cultivars.

### Metabolic network analysis among the eight grape varieties shows a strong structuring of polyphenol metabolism

**Figure 8:**
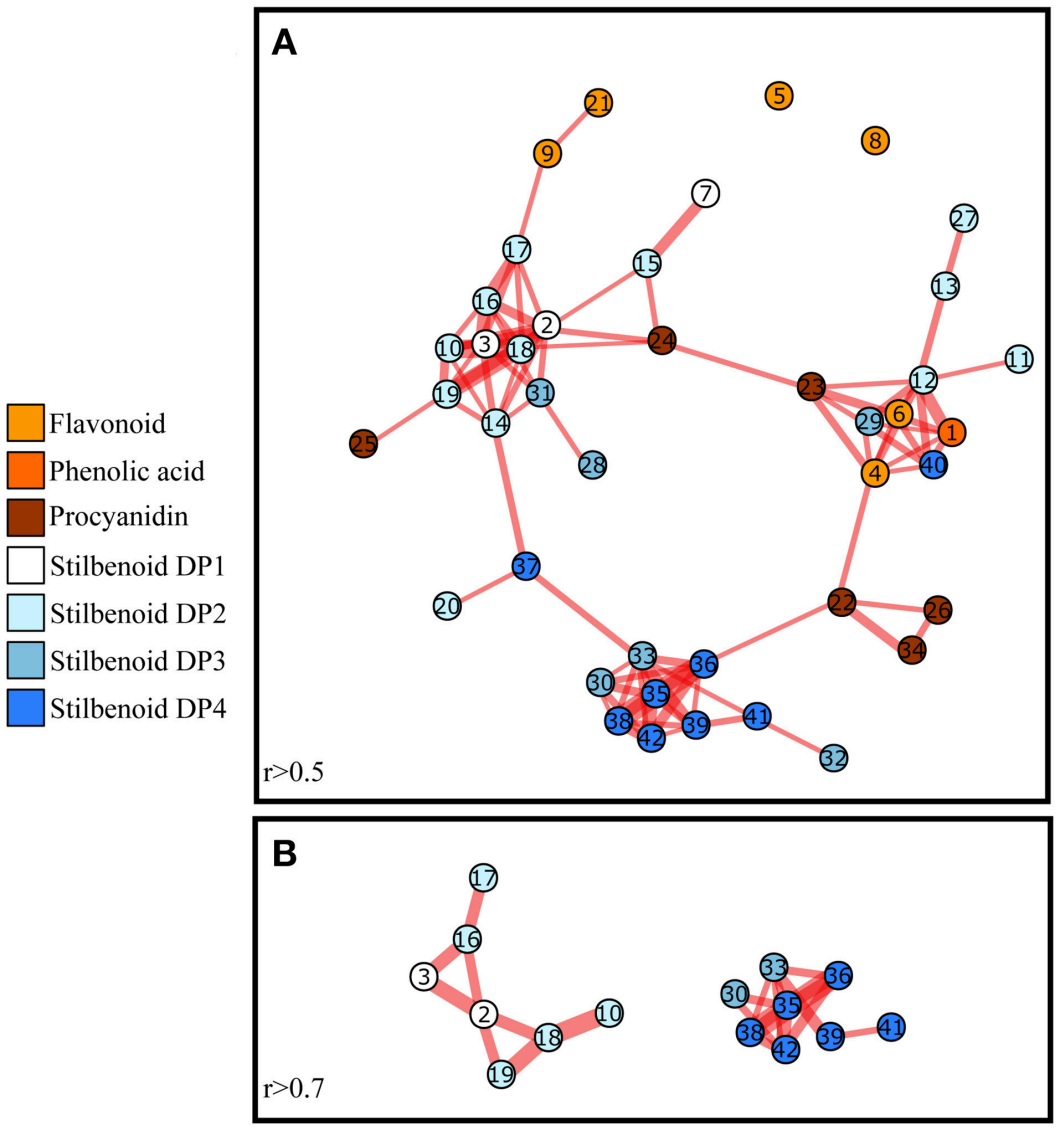
Correlation-based networks of polyphenol metabolism related compounds in grape stems in 2013. Metabolite pairs whose correlation was significant with a minimum correlation coefficient of 0.5 **(A)** and 0.7 **(B)** are connected. Nodes represent metabolites and color refers to the class of polyphenols. Node numbers indicate the ID of the compounds as given in Table 2. Short node distance (Pearson correlation coefficients) and thick lines (*P-*values) indicate high correlation.

A co-variation analysis of metabolites represents a potential source of information on the global properties of the system. The fluctuations of metabolite levels and their interdependence may reveal metabolites or pathways depending on similar control mechanisms including endogenous and exogenous factors (Steuer, 2006). To reveal polyphenols with similar pattern of accumulation among the 8 tested genotypes, pair-wise correlations were calculated between metabolites levels within the dataset of 42 compounds in year 2013. Significant correlations were visualized in a correlation-based network where metabolites are represented by node and significant correlation by edges (Figure 8). Among the 861 tested pair-wise correlations, 68 significant positive correlations were found (threshold: *r* > 0.5 and *P-*value < 0.05). The correlation network showed that the structurally related compounds are intercorrelated and clustered together (Figure 8A). On the whole, metabolites belonging to flavonoid or stilbenoid pathways are grouped together within the network. The clustering topology of the network allows the identification of groups of nodes with similar pattern of behaviors among the eight tested genotypes. One group of nodes mainly containing monomers (*E*-resveratrol, *E*-piceatannol) and dimers (scirpusin A1 and A2, restrytisol A and B) showed a higher degree of node-connectivity (threshold: *r* > 0.7 and *P-*value < 0.05; Figure 8B). This high connectedness underlines the dominant role of both *E*-resveratrol and *E*-piceatannol as precursor of oligomeric-type stilbenoids (Moss et al., 2013). As an example (Figure 8B), scirpusin A (16), a piceatannol-resveratrol heterodimer is connected by an edge with both resveratrol (2) and piceatannol (3) whereas restrytisol A (18) and B (19) an oxidized resveratrol-resveratrol homodimer is connected with resveratrol (2) only. A second group of nodes belonging to the stilbenoid pathway mainly contained trimers and tetramers of resveratrol with a high degree of node-connectivity. A third group of nodes including both procyanidin and stilbenoid showed also a clustering.

## Discussion

Grape polyphenols play a central role in defense traits and additionally hold numerous health-benefit effects. In the present study, we investigate the metabolic phenotype of *V. vinifera* L. stems from a field experiment including eight varieties, to assess their polyphenol signature. Since phenylpropanoid metabolism might be influenced by abiotic and biotic stimuli (Vogt, 2010), we designed a field experiment in an attempt to homogenize as much as possible the impact of pedo-climatic factors on plant physiology in order to allow a full expression of the potential genetic determinism of grape phenylpropanoid metabolism. When targeting the analysis on stilbenoids, the major polyphenols accumulated in grape stems, marked differences were observed. Afterwards, extending the analysis to the full polyphenol content, PLS-DA technics enabled the discrimination of polyphenol signatures according to the berry color and the grape variety. HCA strategy based on PLS-DA showed the occurrence of variable discrepancies on polyphenol fingerprints. This highlights the existence of different biochemical distances between the grape varieties, which may unveil genetic backgrounds. In this way, HCA based on 22 microsatellite markers from the eight varieties was generated to figure out the genetic distances. The Fowlkes-Mallows index confirmed a certain degree of similarity between the topology of SSR markers and polyphenol-based dendrograms. Metabolite network analysis showed a fine control of structurally related polyphenols.

### Variation of *E*-resveratrol in *vitis vinifera* L. stems

Previous studies on *V. vinifera* L. stems mainly focused on *E*-resveratrol, as it is historically the most studied stilbenoid compound. We found that *E*-resveratrol levels were genotype dependent and the values were in general agreement with the literature with Pinot noir and Gamay as high *E*-resveratrol producer (Aaviksaar et al., 2003; Püssa et al., 2006; Karacabey and Mazza, 2008; Rayne et al., 2008; Lambert et al., 2013). Nevertheless, *E*-resveratrol content for a same grape variety could significantly vary. In Pinot Noir, Karacabey and Mazza (2008) found various resveratrol levels (from 1,290 to 4,060 mg kg^−1^ DW) depending on extraction procedures and our previous studies reported a 19-fold increase (from 250 ± 112 to 4,762 ± 952 mg kg^−1^ DW) during grape stem storage (Houillé et al., 2015a; Billet et al., 2018). The differences observed among these studies emphasize technical requirement of stably measurable procedures for extraction and storage of grape stems before stilbenoid analysis. Nevertheless, *E*-resveratrol is one stilbenoid among many other polyphenols accumulated in grape stems and we chose a metabolomic approach to explore the metabolic diversity in *V. vinifera* L. stems.

### Polyphenol fingerprint in *vitis vinifera* L. stems

The majority of metabolomic studies on grape and grape-derived products concern the wine for authentication, traceability, and fraud (Alañón et al., 2015) with the development of home-built wine metabolomic databases (Vaclavik et al., 2011; Roullier-Gall et al., 2014) and commercialized package (Wine screener by Bruker). However, wine production starts with vine growing and only few studies harness metabolomics-based characterization of grapevine (Son et al., 2009). In a promising approach, Son et al. (2014) described the metabolic fingerprints of six different grape cultivars grown under greenhouse conditions using skin and pulp and NMR-based metabolomics coupled to PCA analysis. Recently, metabolomics based on vineyard experiments coupled to multivariate statistical analyses has proven to be a powerful approach to identify biomarkers during berry development (Zamboni et al., 2010; Wang et al., 2017) and in *Botrytis cinerea*-infected berries (Agudelo-Romero et al., 2015; Negri et al., 2017). Nevertheless, the influence of variety on grape metabolome in a vineyard experiment has never been reported. In the present study, we assess the genotype signature in stems of 8 grape varieties in a vineyard experiment by UPLC-MS-based metabolomic approach coupled to multivariate statistics. The UPLC-MS-based targeted metabolomic method enabled a one-shot analysis of 42 metabolites including 16 analytes identified by comparison with authentic standards and 26 putatively identified from literature. Stilbene-type polyphenols (29/42) prevailed in grape stems compared to flavonoid-type (Figures 5C,D) suggesting a predominance of the stilbene synthase (STS) branch over the chalcone synthase (CHS) branch in wood tissues (Figure 1). It thus contrasts with the polyphenol composition of berries where chalcone derivative products are more abundant (Yin et al., 2016). STS and CHS, two similar polyketide synthases, compete for the same substrate and a negative correlation between the two branches has been already suggested (Jeandet et al., 1995). Since the metabolic flux of polyphenols is preferentially oriented toward the CHS branch in berries, the diversity of STS-derivated polyphenols is limited to several monomers including resveratrol, piceid, and pterostilbene (Suzuki et al., 2015). In contrast, in grape stems, the STS branch seems to predominate and the biosynthesis of a large set of resveratrol derivatives is observed. This specific regulation of polyphenol metabolism toward stilbenoid production in woody tissues offers unprecedented opportunities to establish metabolic fingerprints of *V. vinifera* L. genotypes.

### Metabolic profiles of grape stems enable discrimination according to berry skin color

At a first level, the present polyphenol-targeted metabolomics of grape stems coupled to MVA enables the discrimination of genotypes according to the cultivar color. In berries, anthocyanins are accumulated during ripening giving the characteristic red color of skin. The assessment of berry metabolic fingerprints from a large germplasm collection highlights kaempferol and quercetin as biomarkers of white cultivars and B-ring trihydroxylated anthocyanins as biomarkers of the red cultivars (Mattivi et al., 2006). Although anthocyanins are not accumulated in stems (Püssa et al., 2006), the present stem-based metabolomic study showed that it is possible to discriminate color variety without berry. Interestingly, a group of stilbenoid DP4 (peak 35–39, 42) was predominantly accumulated in the stems of the red varieties in 2013 and 2017. Very few studies raised the question of stem-originated polyphenols as biomarkers of berry color (Figure 4). A comparison of the stilbenoid composition (*E*-resveratrol, *E*-piceatannol, *E*-ε-viniferin, miyabenol C, vitisin B, and hopeaphenol) of 16 varieties suggested that red and white varieties share a common stem metabolic fingerprint (Lambert et al., 2013). In a study performed on 8 Portuguese varieties, the stems of the 3 red varieties were globally richer in epicatechin, quercetin-3-O-rutinoside and caftaric acid whereas the 3 white varieties accumulated more *E*-ε-viniferin (Barros et al., 2014). The present study showed that a metabolic profile based on a large number of metabolites coupled to PLS-DA discriminates of two distinct fingerprints with DP4 stilbenoids as biomarkers of the red varieties, however future experiments should confirm such trends in a large germplasm collection.

### Metabolic profiles of grape stems enable discrimination according to genotype

Beyond the classification depending on berry skin color, metabolic profiles of grape stems enabled discrimination of varietal origin suggesting that polyphenol profiles exhibit variety-dependent metabolic fingerprints (Figure 5). HCA perfectly separated the five genotypic replicates but also grouped genotypes according to their biochemical distances, thus suggesting a classification in accordance with their genetic distance. This chemotaxonomic notion was tested by comparing clustering similarities of both metabolomic (42 grape stem polyphenols) and genotypic (22 SSR neutral markers) datasets using the Fowlkes-Mallows index. The cut-off levels corresponding to cluster numbers between 3 and 5 gave a high value for the Fowlkes-Mallows index, indicating a greater similarity between genotype and biochemical distances. A first group of metabotypes (Gamay, Chardonnay, and Pinot Noir) corresponds to closely related genotypes previously grouped called “Noirien” when defined as eco-geographical groups by ampelographers (Levadoux, 1948; Bisson, 1999). A second group of metabotypes (Sauvignon and Chenin) belongs to the group called “Messile” originated in the French Loire Valley. Recently, these parentage relationships were confirmed by DNA sequence studies using SSR and SNP markers (Cipriani et al., 2010; Myles et al., 2011; Lacombe et al., 2013). Chardonnay and Gamay are full-siblings of Pinot noir and Gouais Blanc and Chenin is a possible natural cross of between Sauvignon and Savagnin. The biochemical distance of Cabernet Franc metabotype from “Messile” and “Noirien” groups was coherent with its genetic distance, since Cabernet Franc belongs to the “Carmenet” group and is related with cultivars from Basque Country (Boursiquot et al., 2009). Surprisingly, the clustering of Malbec metabotype within the “Messile” group (Savagnin and Chenin) does not match its genetic origin, since Malbec (syn: Cot) is a result of a cross between Prunelard and Magdeleine Noire des Charentes (Boursiquot et al., 2009). We also noted that in the second dataset (year 2017) the Gamay metabotype became irregularly distant from its genetic group (Chardonnay and Pinot Noir). Genotype-environment interactions may explain the differences observed between metabolomic and genetic distances and the vintage variations (Carreno-Quintero et al., 2013) showing that polyphenol signature is the expression of a genetic determinism on a given “terroir” (Anesi et al., 2015).

### Advantages and limits of field-based metabolomic studies

Field-based metabolomic experiments may reflect real growth conditions, however such investigations are challenging because the metabotype reflects both genetic and environmental conditions. In the present field experiment, the genotypic discrimination was achieved using a harvest protocol that limits the effect of environmental conditions and highlights variations due to the genotype. Because, polyphenol content in grape stems might be locally induced in response to pathogen attacks (Houillé et al., 2015b), the quality of sampling design in the vineyard is determinant to limit intra-plot variations. For metabolomic studies on grape stems, the large sampling size in the vineyard (60 stalks/plot/variety) combined to a randomization step (mixing of cut stalks from one plot and constitution of 5 replicates) overcomes these variations. Similarly, for metabolomic studies on grape berries grown in vineyards, crucial steps of sampling have been also characterized. Since sunlight shapes the spatial pattern of metabolites inside grape cluster (Reshef et al., 2017), this variation needs to be taken into account with adapted sampling protocols. Dealing with the different sources of variability in real growing conditions (genotypes, environment, and management interventions) constitutes one of the future challenges for field-omics (Alexandersson et al., 2014; Anesi et al., 2015).

### Metabolic network correlation suggests a fine control in polyphenol accumulation

The knowledge on biochemical pathways that govern resveratrol oligomerization remains largely limited (Chong et al., 2009). The identification of groups with similar regulatory mechanisms can thus be used to generate new hypotheses on corresponding biochemical pathways (Steuer et al., 2003; Toubiana et al., 2013). For instance, the identification of different clusters within stilbenoid nodes underlies the presence of different regulatory mechanisms within stilbenoid metabolism for several monomers and dimers in one hand and for trimers and tetramers in the other hand. The presence of a third group of nodes, including both procyanidins and stilbenoids, suggests that polyphenols from both flavonoid and non-flavonoid pathways might be governed by similar regulatory mechanisms potentially involving common oxidative polymerization enzymes or transcription factors.

## Conclusions

We developed a UPLC-MS-based targeted metabolomic tool enabling the discrimination of grape stem metabotypes according to the variety in a field experiment. The inter-varietal distances based on biochemical composition suggested a relation with the known genetic distances. The metabolite network correlation revealed the interdependence of certain metabolites within polyphenol metabolism regulated by similar control mechanisms. In a near future, the present polyphenol metabotyping approach coupled to multivariate statistical analyses might assist grape selection programs to improve metabolites with health-benefit potential and plant defense traits.

## Author contributions

KB and BH developed the UPLC-MS methods and performed the analyses. LG, MC, and NG-G were involved in the study design. TD supervised the statistical analysis. GD, SB, AO, and VC carried out data collection and analyses. AL conceived, supervised this study and wrote the first draft of the manuscript; all authors were involved in writing, and gave final approval for publication.

### Conflict of interest statement

The authors declare that the research was conducted in the absence of any commercial or financial relationships that could be construed as a potential conflict of interest.
