# Supplementary material for: Field-Based Metabolomics of Vitis vinifera L. Stems Provides New Insights for Genotype Discrimination and Polyphenol Metabolism Structuring
Source: Front Plant Sci. 2018 Jun 21;9:798. doi: 10.3389/fpls.2018.00798 (PMC6021511; doi:10.3389/fpls.2018.00798)
Supplement: Supplementary file 1 [file Table_1.docx]

Table S1: Accurate quantification of stilbenoid in grape stems from studied cultivars.

|  | stilbenoid concentration (mg kg^-1^ DW) | | | | | | | |
| --- | --- | --- | --- | --- | --- | --- | --- | --- |
| cultivar | *E*-resveratrol | piceatannol | *E*-ε-viniferin | ampelopsin A | *E*-miyabenol C | hopeaphenol | isohopeaphenol | *E*-vitisin B |
| Sauvignon | 804 (366) | 162 (81) | 2673 (1319) | 840 (433) | 90 (45) | 1812 (955) | 276 (136) | 646 (335) |
| Chenin | 1625 (587) | 277 (133) | 2667 (1190) | 308 (164) | 67 (40) | 2116 (975) | 244 (155) | 205 (93) |
| Chardonnay | 2859 (270) | 353 (65) | 3033 (318) | 2533 (299) | 72 (13) | 2606 (516) | 560 (123) | 160 (45) |
| Pinot Noir | 2615 (391) | 315 (92) | 3537 (611) | 1449 (354) | 110 (23) | 2411 (696) | 645 (176) | 491 (131) |
| Grolleau | 1243 (530) | 220 (73) | 3004 (433) | 349 (115) | 78 (12) | 7037 (1562) | 614 (154) | 368 (142) |
| Gamay | 2923 (906) | 382 (111) | 2808 (371) | 3679 (453) | 87 (13) | 3466 (583) | 845 (265) | 773 (133) |
| Malbec | 1869 (625) | 381 (84) | 3352 (488) | 395 (68) | 52 (17) | 1277 (276) | 303 (87) | 41 (13) |
| Cabernet franc | 2494 (521) | 472 (84) | 1932 (193) | 894 (132) | 71 (30) | 4103 (580) | 744 (465) | 388 (109) |
| Means | 2054 (788) | 320 (99) | 2876 (491) | 1306 (1212) | 78 (17) | 3103 (1815) | 529 (228) | 384 (248) |
